# Characteristics of Young Adults with Autism Spectrum Disorder Performing Different Daytime Activities

**DOI:** 10.1007/s10803-018-3730-7

**Published:** 2018-08-27

**Authors:** Ane Knüppel, Gry Kjærsdam Telléus, Helle Jakobsen, Marlene Briciet Lauritsen

**Affiliations:** 1 Aalborg University Hospital, Psychiatry, Mølleparkvej 10, 9000 Aalborg, Denmark; 2 Department of Clinical Medicine, Aalborg University, Søndre Skovvej 15, 9000 Aalborg, Denmark

**Keywords:** Autism spectrum disorder, Education, Occupation, Daytime activity, Schooling

## Abstract

Daytime activity, in terms of engagement in an occupation or education, is highly important for individuals with autism spectrum disorder (ASD), regardless of their level of functioning. In this nationwide survey, the parents of young adults diagnosed with ASD in childhood (n = 1266) provided information about the current daytime activity of their child, as well as behavioral characteristics, comorbidity, history of schooling during primary and secondary school, and availability of support. The young adults without a regular daytime activity constituted approximately one-fifth of the sample and had more behavioral difficulties and comorbidities than young adults with a daytime activity. Intellectual disability, part-time job, history of schooling, including type of school, and availability of support were found to be associated with daytime activity.

## Introduction

Daytime activities for adolescents and adults with autism spectrum disorder (ASD) have been investigated since the earliest ASD adult outcome studies of the 1960s (Rutter et al. 1967). In these studies, which often rely on smaller samples, rates of the study population in paid employment vary considerably, from none to approximately half of the population (Barneveld et al. 2014; Billstedt et al. 2011; Gillespie-Lynch et al. 2012; Ruble and Dalrymple 1996; Wolf and Goldberg 1986). However, rates are difficult to compare owing to not only a change in the concept of ASD over the years but also a shift in the state of the markets, which affects the possibilities of finding and sustaining employment. Furthermore, enrollment in postsecondary education is an alternative to employment for young adults with ASD, especially in more recent studies (e.g., Taylor and Seltzer 2012), which means that young adults with ASD may be classified as being in education instead of in employment for a longer period. In addition to being regularly employed or attending education, some individuals with ASD have occupations in different kinds of supported or sheltered settings. In some studies, most of the people studied had work in such settings (Gray et al. 2014; Howlin et al. 2004). Furthermore, studies have found that a considerable proportion of individuals with ASD do not have any regular daytime activity, but the proportion is found to vary widely, from approximately ≤ 10% (Farley et al. 2009; Gray et al. 2014; Taylor and Seltzer 2012; Venter et al. 1992) to approximately 20–40% (Barneveld et al. 2014; Cederlund et al. 2008; Gillespie-Lynch et al. 2012; Osada et al. 2012). Compared with adolescents with speech and language impairment, learning disability, and intellectual disability (ID), adolescents with ASD were found to have the highest rate of no regular daytime activity in the period after high school (Shattuck et al. 2012).

### Benefits of Having a Daytime Activity

As stated by Hendricks (2010), paid employment has the benefit of, for instance, earning wages to support oneself. However, other benefits are emphasized, such as pursuing one’s own interests, promoting personal dignity, learning new skills, developing social relations, and being able to contribute to society (Hendricks 2010; Holwerda et al. 2013). Results from a survey including the views of adults with ASD supported this notion, with the participants emphasizing the positive aspects of employment, such as providing an opportunity to apply and develop knowledge, skills, and interests, enabling a sense of being accepted and valued, and making a difference in the lives of others (Baldwin et al. 2014). However, it is not only paid, competitive employment that is associated with benefits. García-Villamisar and Hughes (2007) found that adults with ASD in supported employment had improved cognitive performance on, for example, executive tasks compared with those in an unemployed group. Furthermore, Taylor, Smith and Mailick (2014) concluded from their study on adults with ASD that greater vocational engagement was related to subsequent reductions in autism symptoms and maladaptive behavior and to improvements in adaptive functioning. Hence, being in employment seems to be beneficial to adults with ASD, and participation in work-related activities might comprise some of the same positive aspects as paid employment. Thus, having a regular daytime activity should be considered of high importance for adults with ASD (Billstedt et al. 2011; Parsons 2015).

### Factors Associated with Different Daytime Activities

Efforts have been made to clarify factors associated with individuals performing different types of daytime activity. From ASD outcome studies, IQ was found to be associated with occupation (Cederlund et al. 2008; Gray et al. 2014; Howlin et al. 2004; Venter et al. 1992). However, as emphasized by Howlin et al. (2004), the relationship between IQ and outcome is not simple, as absence of ID (i.e., IQ > 70) is associated with a better outcome, while it is not found that the higher the IQ is, the better the outcome (Howlin et al. 2004). In addition, it has been demonstrated that young adults with ASD without ID were approximately three times more likely to have no daytime activity than young adults with ASD and ID (Taylor and Seltzer 2011). Thus, the association between ID and daytime activity is complex. Furthermore, higher functional ability and independence in living and self-care, fewer ASD symptoms, and better social and communicative skills are associated with employment (Carter et al. 2012; Chiang et al. 2013; Holwerda et al. 2013; Roux et al. 2013; Shattuck et al. 2012; Taylor and Seltzer 2011), indicating that employed individuals with ASD generally seem to function well in different aspects.

There are different kinds of education and occupation customized for individuals with disabilities that make a regular daytime activity possible, but less is known about what characterizes individuals performing customized education or occupations or individuals without a regular daytime activity. However, in studies based on samples drawn from the longitudinal studies by Taylor and colleagues (Taylor and Seltzer 2011; Taylor et al. 2015), young adults with ASD performing different types of daytime activity were compared on, for example, functional independence and autism symptoms. It was found that, to some extent, differences in behavioral functioning could be distinguished between groups. For instance, individuals without a regular daytime activity had higher functional independence and fewer ASD symptoms than individuals in day services (e.g., sheltered workshop) but lower functional independence and more ASD symptoms than individuals enrolled in postsecondary education (Taylor and Seltzer 2011). However, the sample sizes in these studies were relatively small (n = 66; n = 73), and more studies are needed to replicate and extend the findings.

Studies also investigated the relationship between daytime activity and contextual factors such as earlier work experiences and schooling in mainstream or special educational settings. As summarized in a review by Test et al. (2009), adults from different disability groups were more likely to be engaged in postsecondary education or employment if they had been in paid employment during high school. The same result was found in a study where individuals with ASD represented approximately one-third of the study sample, which included other disability groups (Carter et al. 2012), but this result could not be confirmed when explored in a sample including only adults with ASD (Chiang et al. 2012). For type of schooling (i.e., mainstream or special educational settings), studies have investigated associations with both secondary/postsecondary education and occupation but with diverse results. For students with different disabilities, a review found that students included in mainstream education were more likely to be engaged in postsecondary education or employment (Test et al. 2009). Some ASD-specific studies have reached the same conclusion (Chan et al. 2017; Chiang et al. 2012; Woodman et al. 2016), while others have not found associations between type of schooling and later education or occupation (Foster and Pearson 2012; Venter et al. 1992).

### Aim of the Present Study

The aim of the present study was to compare groups of young adults diagnosed with ASD in childhood and currently engaged in different types of daytime activity using a large and nationwide sample from the AutCome survey. Hence, groups of individuals with ASD in so-called normative education/occupation, in customized education/occupation, or without regular daytime activity were compared on behavioral parameters such as autism symptomatology, adaptive behavior, ID, maladaptive behavior, and psychiatric comorbidity. Additionally, data on the number of working hours and parental evaluations of their son’s/daughter’s experience of being in an occupation are presented. Furthermore, this study addresses whether contextual factors primarily related to schooling during primary and lower secondary school are associated with type of current daytime activity.

## Methods

### Study Population and Procedure

A Danish nationwide survey, the AutCome study, was conducted in 2016 regarding outcomes for adolescents and adults diagnosed with ASD in childhood (Knüppel et al. 2018b). In the survey, individuals with ASD and their parents were invited via mail to complete a questionnaire about different aspects of outcome, such as adaptive functioning and current daytime activity (more details are provided in Knüppel et al. 2018b). In the present study, only information provided by the parents was used. Owing to the aim of this study, only individuals of at least 18 years of age were selected, as the ordinary age for completing compulsory schooling (i.e., primary and lower secondary school) in Denmark is expected to be exceeded at this age. The study population consisted of young adults with ASD (n = 1266) aged 18–26. They were diagnosed with ASD before the age of 14 at psychiatric hospitals for children and adolescents in Denmark and identified in the Danish Psychiatric Central Research Registry (DPCRR) (Mors et al. 2011). The following ICD-10 autism diagnoses were included: infantile autism (F84.0), atypical autism (F84.1x), Asperger’s syndrome (F84.5), and pervasive developmental disorder, other (F84.8).

National register data were used to search for potential differences between responding and nonresponding families in the survey. Subjects of comparisons were the number of psychiatric hospital visits of the individuals with ASD, including the frequencies of different ICD-10 autism diagnoses applied and the sociodemographics of the individuals with ASD and their parents. Only minor differences were found in the analyses; nevertheless, more socioeconomically advantaged families were more likely to complete the questionnaire (Knüppel et al. 2018b).

### Coding of Current Daytime Activity

Three main groups of current daytime activity were created based on answers from the parents in the questionnaires: (1) a group in normative education or occupation; (2) a group in customized education or occupation; and (3) a group without regular daytime activity. The definitions of the three groups of daytime activity, as well as the coding of the data, are provided below.

To describe current daytime activity regardless of whether it was education or employment, several subcategories were created to capture the diversity of daytime activities of the study population (see also Table 1 for this categorization). The composed categories were devised with inspiration from the vocational index developed by Taylor and Seltzer (2012). However, owing to the nature of the data, it was not possible to simply apply the Taylor and Seltzer index. For the present study, each individual with ASD was coded based on parental scoring regarding education, occupation, or other daytime activity in the questionnaire, in addition to written responses stated in text boxes.

**Table 1:** Types of current daytime activity performed by study participants

Normative education or occupation (n = 567)		Customized education or occupation (n = 430)		No regular daytime activity (n = 269)	
Mean age = 21.64 (SD = 2.32)		Mean age = 21.14 (SD = 2.37)		Mean age = 22.33 (SD = 2.15)	
Age of diagnosis of ASD = 9.60 (SD = 3.01)		Age of diagnosis of ASD = 8.58 (SD = 3.42)		Age of diagnosis of ASD = 9.24 (SD = 3.24)	
83.77% male		80.00% male		80.30% male	

Type of daytime activity	% (n)	Type of daytime activity	% (n)	Type of daytime activity	% (n)
Employment in community without support	19.58% (111)	Employment in community with support	10.70% (46)	No regular activities reported	100.00% (269)
Postsecondary education	26.81% (152)	Sheltered vocational setting	20.93% (90)		
Upper secondary education (including vocational education)	53.62% (304)	Volunteering^a^	0.93% (4)		
		Primary and lower secondary school	12.33% (53)		
		Customized educational program	44.88% (193)		
		Other degree-seeking education	4.88% (21)		
		Other nondegree-seeking education	3.95% (17)		
		Folk high school^a^	1.40% (6)		

*SD* standard deviation, *ASD* autism spectrum disorder

^a^It was not clear from the information made available by the parents whether individuals volunteering or attending folk high schools were performing normative or customized activities, but it was decided to categorize these individuals in the group of customized daytime activity

For ongoing educational programs, seven subcategories were created: (1) postsecondary education; (2) upper secondary education, including vocational; (3) primary and lower secondary education; (4) customized educational program; (5) other degree-seeking educational program; (6) other nondegree-seeking educational program; and (7) folk high school (nonformal adult education). The customized educational program can be described as an upper secondary educational program for individuals with disabilities. The content of this program differs across educational institutions and can be tailored to the needs of the individual student. For occupation, four categories were created: (1) employment in community without support; (2) employment in community with support; (3) sheltered vocational setting; and (4) volunteering. For all categories, there were no minimum requirements about number of hours engaged in an occupation or in an educational program. This implied, for example, that individuals enrolled in upper secondary education on a part-time basis were categorized as attending upper secondary education along with full-time students. Furthermore, a category was created for individuals with no participation in educational programs or occupational activities.

The three main groups of current daytime activity used in the present study were defined as follows (Table 1): (1) the group of normative education or occupation was composed of adults coded with the following types of daytime activity—employment in community without support, postsecondary education, and upper secondary education, including vocational; (2) the group of customized education or occupation was composed of adults coded with the remaining educational and occupational categories; and (3) the group without regular daytime activity was composed of adults with no participation in educational programs or occupational activities.

Note that for individuals in normative education or occupation, some degree of support or services might be given; however, it was decided to categorize all individuals attending ordinary educational programs or engaged in ordinary occupation accordingly. Furthermore, for all individuals with ASD engaged in vocational activities, parents were asked to state working hours per week and to evaluate how suitable the vocational activity was for their son/daughter: Evaluation of the fit between the occupation and the level of education of their son/daughter (yes; no, he/she is overqualified; no, he/she is underqualified); evaluation of whether their son/daughter likes the occupation (most of the time; sometimes; never/almost never); evaluation of the fit between the occupation and the interests of their son/daughter (most of the time; sometimes; never/almost never); the opportunity for their son/daughter to use their skills in the occupation (most of the time; sometimes; never/almost never); experience of success in the occupation (most of the time; sometimes; never/almost never); whether the place of occupation shows consideration for eventual disabilities (most of the time; sometimes; never/almost never; not necessary); and whether organized support exists at the place of occupation (yes; no). The response “do not know” was an option for every question asked.

### Survey Data Used for Comparisons of Daytime Activity Groups

Information on behavioral data and comorbidity (adaptive behavior, autism symptomatology, maladaptive behavior, psychiatric comorbidity, and ID) obtained from the survey was used for comparisons across groups of daytime activity. For analyses of factors associated with group of daytime activity, data concerning schooling during primary and lower secondary school, adequacy/availability of support and services, ID, and part-time job were used. The parents provided all data.

The Adaptive Behavior Assessment System-II (ABAS-II) was applied for assessment of adaptive behavior and skills (Harrison and Oakland 2004). The adult form (aged 16–89 years) was used, yet the optional domain in the scale (work) was not applied in this study. Raw scores were converted to the General Adaptive Composite Score (GAC), which has a mean of 100 (SD = 15); a higher score indicates better adaptive behavior. Ordinal alpha was calculated for each domain in the ABAS-II and found to be in the range of 0.961–0.986, indicating excellent internal consistency within each domain.

The Ritvo Autism and Asperger Diagnostic Scale (RAADS)-14 Screen was used to assess the presence and extent of autistic symptoms. The RAADS-14 Screen is a three-domain scale for the screening of autistic symptoms based on the RAADS-Revised (Eriksson et al. 2013). The RAADS-14 Screen contains 14 items scored on a 0–3 Likert scale. It was originally constructed as a self-report instrument, but owing to the need of a short scale in the survey, it was applied as a parental report. It has been tested in a small sample of parents of adults with ASD with good results (J.M. Eriksson, personal communication, May 16, 2015). A higher score indicates more ASD symptoms. Ordinal alpha was calculated for each domain in the RAADS-14 Screen (0.674, 0.720, and 0.882), indicating acceptable internal consistency overall.

Maladaptive behavior was coded as two separate binary variables (present vs. not present): current maladaptive behavior and lifetime maladaptive behavior, covering the presence of maladaptive behavior at any time in life. Behavior classified as self-destructive, including breaking belongings and being defiant, disruptive, hurtful to others, and/or socially offensive, was coded as the presence of maladaptive behavior and was rated as present by the parents if the existence of these behaviors constituted difficulties in everyday life for their son/daughter with ASD or the surroundings.

Furthermore, parents rated current psychiatric comorbidity. In the survey, they could either mark several common psychiatric diagnoses or write the diagnosis/diagnoses themselves. For each diagnostic category, a binary variable was created (present vs. not present) in addition to coding overall current psychiatric comorbidity as a binary variable (no current psychiatric comorbidity vs. current psychiatric comorbidity). Presence of ID was rated by parents and coded as a binary variable (present vs. not present). Information on having a part-time job at any point in addition to attending school/enrollment in education was coded as a binary variable (yes vs. no).

With respect to the schooling of their child with ASD during primary and lower secondary school, parents marked the type of schooling for each grade (first to ninth/tenth grade). These data were categorized for each year of school according to three groups: (1) mainstream education; (2) special education (generic); and (3) special education (ASD specific). A variable called compulsory schooling was created to classify the primary type of school attended, where the adults with ASD were classified as primarily educated in mainstream education or primarily educated in special education (generic or ASD specific) according to years spent in each of the two types of educational settings. Furthermore, another variable, called schooling (hierarchical), was created to classify individuals according to the following types of school ever attended, hierarchically: mainstream education if never been in special education; generic special education if never been in educational settings specific for children with ASD; and special education specific for children with ASD, if ever attended. A third variable was also created for school type of completed lower secondary school with the three categories applied: (1) mainstream education; (2) special education (generic); and (3) special education (ASD specific). Number of school changes during primary and lower secondary school was categorized into three groups: (1) no changes of school; (2) 1–2 changes of school; and (3) ≥ 3 changes of school.

Adequacy of support/services during lower secondary school was evaluated by parents and categorized into two groups: (1) support/services adequate or not necessary and (2) support/services never or rarely adequate. Availability of current support/services to the individual with ASD and/or the family was also evaluated by the parents and categorized into three groups: (1) support/services available; (2) support/services not available but needed; and (3) support/services not necessary.

### Register Data Used for Comparisons of Daytime Activity Groups

Information regarding individuals with ASD (age, sex, age of diagnosis of ASD, municipality of residence) and their parents (highest completed education) was derived from the Danish national registers to describe the groups of daytime activity and analyze factors associated with each group of daytime activity. Age of diagnosis of ASD was defined as the age of the individual when the (first) diagnosis of ASD in the DPCRR was registered. Data (from 2014) regarding municipalities were collected as density of population (Statistics Denmark, n.d.) and were grouped into (1) densely populated area, (2) intermediate population area, and (3) thinly populated area. For highest completed parental education, data (from 2014) were grouped as (1) primary and lower secondary school, (2) upper secondary education, or (3) qualifying education (postsecondary or vocational education).

### Ethics

The study was registered with the Danish Data Protection Agency (record no. 2008-58-0028), and the Danish Health Data Authority provided the mailing addresses used to invite the study population. The families invited were given thorough, written information about the study, including a statement that participation was voluntary. Data were anonymized after collection.

### Statistical Analysis

Descriptive analyses of the sample were performed divided into groups based on current daytime activity. Internal consistency of the scales was estimated by calculation of the ordinal version of Cronbach’s alpha (Gadermann et al. 2012). Missing values for the ABAS-II and RAADS-14 Screen were handled with multivariate imputation using chained equations, with five imputations for each missing value. For the ABAS-II, a maximum of two missing values in each domain was accepted for every respondent, and for the RAADS-14 Screen, one missing value in total was accepted. For other variables, no imputations were made, resulting in a variable number of observations in the analyses performed. Therefore, the sample size (n) for each analysis is specified.

For comparisons of groups, Fisher’s exact tests or linear regression analyses were performed, whichever fitted the data. Cramer’s *V* was used for estimation of effect size with the descriptors of magnitude provided by Cohen (1988). Group differences were based on a significant *p*-value (Fisher’s exact test, *p* < 0.05) and a minimum small effect size (Cramer’s *V* ≥ 0.10 or ≤ − 0.10, degrees of freedom = 1). For regression analyses, group differences were based on significant results (*p* < 0.05).

Multinomial logistic regression analyses were performed with one independent variable at a time with adjustment for age and sex of the individuals with ASD in each analysis. The result of a multinomial regression analysis, a relative risk ratio (RRR), is commonly interpreted as an odds ratio (UCLA: Statistical Consulting Group, n.d.). Post hoc Wald tests were conducted for comparisons of RRR within each analysis. Significance was set as *p* < 0.05. Data analyses were performed using SPSS Statistics version 24 (IBM Corp. 2016), STATA version 14.2 (StataCorp. 2015), and R version 3.2.5 (R Core Team 2016).

## Results

### Description of Daytime Activity Groups

Types and proportions of current daytime activities for the entire sample are shown in Table 1.

Overall, adults with ASD in normative education/occupation constituted 44.8% (n = 567/1266) of the total sample, while adults with ASD in customized education/occupation represented 34.0% (n = 430/1266), and adults with ASD without regular daytime activity represented 21.2% (n = 269/1266). The majority of the individuals with ASD in normative education/occupation were enrolled in upper secondary education (53.6%, n = 304/567). Almost half of the group of adults in customized education/occupation were attending a customized educational program (44.9%, n = 193/430), and approximately one-fifth were in sheltered vocational settings (20.9%, n = 90/430).

For adults with ASD engaged in any type of occupation, the majority worked at least 30 h/week (41.1%; n = 94/229) or between 15 and 29 h/week (33.2%; n = 76/229). A further 16.6% (n = 38/229) worked < 15 h/week, and 9.2% (n = 21/229) worked varying h/week, had seasonal work, or their parents were unsure about their child’s exact working hours. The parental evaluation of their son’s/daughter’s experience of being in an occupation is shown in Table 2.

**Table 2:** Experiences of individuals with ASD engaged in vocational activities according to parental evaluations

	% (n)
Fit between occupation and level of education, *% yes*	80.60% (187)
Liked the occupation, *% most of the time*	90.00% (207)
Fit between occupation and own interests, *% most of the time*	63.20% (146)
Opportunity to use own skills in occupation, *% most of the time*	64.35% (148)
Experience of success at occupation, *% most of the time*	72.29% (167)
Place of occupation took into account potential disabilities	
*% most of the time*	51.30% (118)
*% not necessary*	27.83% (64)
Organized support at place of occupation, *% yes*	47.62% (110)

Included individuals employed in the community with/without support, individuals in sheltered vocational settings, and individuals volunteering

Total n varies between 230 and 232

*ASD* autism spectrum disorder

The majority of the parents (80.6%, n = 187/232) considered that there was a fit between type of occupation and educational level of the adults with ASD and that the adults with ASD liked their occupation in most cases (90.0%, n = 207/230). Approximately half of the adults with ASD in an occupation (47.6%, n = 110/231) experienced organized support at the place of occupation.

### Comparisons of Daytime Activity Groups

The three groups of daytime activity were compared across behavioral parameters; the results are shown in Table 3 (continuous variables) and 4 (categorical variables).

**Table 3:** Comparisons of current daytime activity groups: continuous variables

	Group 1 normative Mean [95% CI] (n)	Group 2 customized Mean [95% CI] (n)	Group 3 none Mean [95% CI] (n)	Regression 1^a^ Group number; coefficient (*p*)	Regression 2^a^ Group number; coefficient (*p*)	Group differences*
Autism symptomatology (*RAADS-14 Screen*)	19.82 [19.00, 20.65] (512)	27.87 [27.00, 28.73] (392)	27.88 [26.75, 29.01] (242)	1; − 8.05 (< 0.001) 2; − 0.01 (0.987)	2; 8.04 (< 0.001) 3; 8.05 (< 0.001)	1 − (2;3)
Adaptive behavior (*ABAS-II, GAC*)	95.10 [93.53, 96.67] (425)	73.51 [71.44, 75.58] (318)	72.80 [70.28, 75.32] (192)	1;22.30 (< 0.001) 2; 0.71 (0.659)	2; − 21.59 (< 0.001) 3; − 22.30 (< 0.001)	1 − (2;3)

Group 1: normative education/occupation; group 2: customized education/occupation; group 3: no regular daytime activity; ABAS-II GAC: adaptive behavior assessment scale II: general adaptive composite score; RAADS-14 Screen: Ritvo Autism Asperger Diagnostic Scale-14 Screen; CI: confidence interval

^a^Linear regression analyses with the RAADS-14 Screen total score or ABAS-II GAC as outcome. For regression 1, group 3 is the reference group. For regression 2, group 1 is the reference group. For the RAADS-14 Screen, 0.28% of the total values were imputed, and for the ABAS-II, 0.56% of the total values were imputed

*Based on regression analyses with significant coefficient (*p* < 0.05)

In terms of the presence and extent of autistic symptoms, the adults in normative education/occupation had a significantly lower mean score on the RAADS-14 Screen than those in the other groups (mean = 19.8, *p* < 0.001). For adaptive behavior, adults with ASD in normative education/occupation had a significantly higher mean score on the ABAS-II than those in the other groups (mean = 95.1, *p* < 0.001). Adults with ASD in customized education/occupation and adults with ASD without regular daytime activity did not differ significantly on autism symptomatology or adaptive behavior (Table 3).

With respect to presence of ID in adults with ASD, all groups differed in the pairwise comparisons (Table 4). The highest proportion of ID was found in the group of individuals in customized education/occupation (33.4%) and the lowest proportion in the group engaged in normative education/occupation (2.6%). Maladaptive behavior was compared according to current presence of maladaptive behavior and lifetime maladaptive behavior. All groups showed high proportions of lifetime maladaptive behavior (61.7–74.3% across groups), with the highest proportion in adults without a regular daytime activity, showing a difference compared with the adults in normative education/occupation. For current maladaptive behavior, proportions were found in the range of 16.1–44.3% and differed across groups. The highest proportion was found for individuals without regular daytime activity. When comparing the percentage of any current psychiatric comorbidity, a difference was found for the adults in normative education/occupation compared with those in the remaining groups. For all groups, approximately half the adults with ASD did not have any current psychiatric comorbidity (45.5–67.5% across groups). With regard to proportions of anxiety and depression, the individuals without regular daytime activity differed from those in the two remaining groups, with higher proportions of anxiety (19.6%) and depression (17.9%) than the adults in normative education/occupation (anxiety 5.7%; depression 5.5%) and the adults in customized education/occupation (anxiety 10.8%; depression 8.7%).

**Table 4:** Comparisons of current daytime activity groups: categorical variables

	Group 1 normative % (n)	Group 2 customized % (n)	Group 3 none % (n)	Group 1 versus 2 p* (Cramer’s *V*)	Group 1 versus 3 p* (Cramer’s *V*)	Group 2 versus 3 p* (Cramer’s *V*)	Group differences**
Intellectual disability, *% yes*	2.60 (14)	33.41 (138)	18.94 (50)	< 0.001 (0.42)	< 0.001 (− 0.28)	< 0.001 (0.16)	1 − 2 − 3
Maladaptive behavior							
Current, *% yes*	16.12 (84)	32.58 (130)	44.31 (109)	< 0.001 (0.19)	< 0.001 (− 0.30)	0.003 (− 0.12)	1 − 2 − 3
Lifetime, *% yes*	61.70 (319)	69.35 (276)	74.29 (182)	0.017 (0.08)	0.001 (− 0.12)	0.209 (− 0.05)	1 − 3
Current psychiatric comorbidity							
No	67.46 (342)	53.73 (209)	45.53 (107)	< 0.001 (0.14)	< 0.001 (− 0.21)	0.048 (− 0.08)	1 − (2;3)
AD(H)D	15.19 (77)	21.59 (84)	17.02 (40)	0.014 (0.08)	0.518 (− 0.02)	0.179 (0.06)	–
Tourette/tics	2.17 (11)	4.37 (17)	4.26 (10)	0.080 (0.06)	0.151 (− 0.06)	1.000 (0.00)	–
Learning disability	4.54 (23)	6.43 (25)	5.11 (12)	0.233 (0.04)	0.713 (− 0.01)	0.601 (0.03)	–
Anxiety	5.72 (29)	10.80 (42)	19.57 (46)	0.006 (0.09)	< 0.001 (− 0.21)	0.003 (− 0.12)	(1;2) − 3
Depression	5.52 (28)	8.74 (34)	17.87 (42)	0.064 (0.06)	< 0.001 (− 0.20)	0.001 (− 0.14)	(1;2) − 3
OCD	3.75 (19)	5.66 (22)	10.64 (25)	0.198 (0.05)	< 0.001 (− 0.14)	0.028 (− 0.09)	1 − 3
Eating disorder	1.97 (10)	1.80 (7)	2.98 (7)	1.000 (− 0.01)	0.432 (− 0.03)	0.405 (− 0.04)	–
Schizophrenia, psychosis	< 0.59 (< 3)	2.57 (10)	4.68 (11)	0.001 (0.11)	< 0.001 (− 0.17)	0.173 (− 0.06)	1 − (2;3)
Bipolar disorder	0.00 (0)	< 0.77 (< 3)	1.28 (3)	0.434 (0.04)	0.031 (− 0.09)	0.153 (− 0.06)	–
Attachment disorder	0.00 (0)	< 0.77 (< 3)	< 1.27 (< 3)	0.434 (0.04)	0.317 (− 0.05)	1.000 (− 0.01)	–

*ADHD* attention-deficit (–hyperactivity) disorder, *OCD* obsessive compulsive disorder

*Fisher’s exact test

**Based on significant difference (Fisher’s exact, *p* < 0.05) and a minimum small effect size (Cramer’s *V* ≥ 0.10 or ≤ -0.10, degrees of freedom = 1)

### Factors Associated with Groups of Daytime Activity

Variables of interest divided between the three groups of daytime activity are shown in Table 5. In Table 6, results from the multinomial logistic regression analyses and Wald tests are shown. In all analyses, adjustments were made for age and sex of the individuals with ASD, and the base outcome was defined as the group of adults without a regular daytime activity. Hence, the risk ratio for being in the normative or customized group of daytime activity relative to the group without regular daytime activity was estimated for each independent variable, given that the age and sex of the individuals with ASD are held constant.

**Table 5:** Variables of interest divided between groups based on current daytime activity

	Normative education/occupation % (n)	Customized education/occupation % (n)	No regular daytime activity % (n)
Sex, *male*	83.77 (475)	80.00 (344)	80.30 (216)
Parental highest educational level			
Primary and lower secondary school	3.18 (18)	4.43 (19)	5.95 (16)
Upper secondary school	1.59 (9)	2.10 (9)	3.35 (9)
Postsecondary/vocational education	95.23 (539)	93.47 (401)	90.71 (244)
Had a part-time job at any point	54.27 (305)	22.30 (95)	24.34 (65)
Availability of current support/services			
Available	27.04 (136)	44.59 (169)	45.45 (110)
No, but would like support	22.27 (112)	29.55 (112)	38.43 (93)
Not necessary	50.70 (255)	25.86 (98)	16.12 (39)
Population density			
Thin	26.81 (152)	36.74 (158)	33.09 (89)
Intermediate	39.33 (223)	36.05 (155)	40.89 (110)
Dense	33.86 (192)	27.21 (117)	26.02 (70)
Compulsory schooling (primary and lower secondary school)			
Primarily in special education	38.88 (208)	74.57 (302)	63.01 (155)
Primarily in mainstream education	61.12 (327)	25.43 (103)	36.99 (91)
Schooling (hierarchical)			
Mainstream	34.11 (175)	5.29 (21)	11.20 (27)
Special, generic	15.98 (82)	21.16 (84)	20.75 (50)
Special, autism	49.90 (256)	73.55 (292)	68.05 (164)
Completion of lower secondary school			
From special education, generic	14.21 (57)	25.66 (88)	18.13 (35)
From special education, autism	31.42 (126)	67.06 (230)	63.73 (123)
From mainstream education	54.36 (218)	7.29 (25)	18.13 (35)
Number of changes of school (during primary and lower secondary school)			
No changes	19.25 (103)	28.64 (116)	17.48 (43)
1–2	61.12 (327)	51.85 (210)	52.44 (129)
≥ 3	19.63 (105)	19.51 (79)	30.08 (74)
Adequacy of support/services in school (during lower secondary school)			
Adequate or not necessary	63.72 (353)	63.08 (270)	53.58 (142)
Never or rarely adequate	36.28 (201)	36.92 (158)	46.42 (123)

**Table 6:** Risk ratio for being in the normative or customized group of current daytime activity relative to the group without regular daytime activity (separate multinomial logistic regression models with no regular daytime activity as base outcome)

	Normative education/occupation				Customized education/occupation				n	Wald test on coefficients for normative versus customized	
	RRR	SE	*p*	95% CI	RRR	SE	*p*	95% CI		chi2(1)	*p*
Parental highest education											
Primary and lower secondary school	0.52	0.18	0.064	[0.26, 1.04]	0.75	0.27	0.415	[0.37, 1.50]	1264	1.19	0.276
Upper secondary school	0.44	0.21	0.090	[0.17, 1.14]	0.61	0.30	0.310	[0.23, 1.59]		0.45	0.503
Intellectual disability											
Present	0.12	0.04	< 0.001	[0.06, 0.22]	2.22	0.43	< 0.001	[1.52, 3.25]	1216	101.90	< 0.001
Had a part-time job at any point											
Yes	3.67	0.61	< 0.001	[2.64, 5.09]	0.89	0.17	0.536	[0.62, 1.29]	1255	95.73	< 0.001
Availability of current support											
Yes	0.19	0.04	< 0.001	[0.13, 0.29]	0.63	0.15	0.046	[0.40, 0.99]	1124	51.01	< 0.001
No, but would like support	0.18	0.04	< 0.001	[0.11, 0.28]	0.47	0.11	0.001	[0.29, 0.75]		28.55	< 0.001
Population density											
Intermediate population density	0.70	0.13	0.052	[0.49, 1.00]	0.79	0.16	0.229	[0.53, 1.16]	1266	0.54	0.463
Thinly populated	0.58	0.11	0.005	[0.39, 0.85]	0.96	0.20	0.855	[0.64, 1.44]		9.60	0.002
Compulsory schooling											
Primarily in special education	0.38	0.06	< 0.001	[0.28, 0.52]	1.89	0.34	< 0.001	[1.33, 2.69]	1186	117.80	< 0.001
Schooling (hierarchical)											
Special, generic	0.26	0.07	< 0.001	[0.15, 0.45]	2.27	0.78	0.018	[1.15, 4.47]	1151	59.75	< 0.001
Special, autism	0.25	0.06	< 0.001	[0.16, 0.39]	2.44	0.76	0.004	[1.33, 4.48]		85.41	< 0.001
Completion of lower secondary school											
From special education, generic	0.27	0.08	< 0.001	[0.16, 0.47]	3.84	1.29	< 0.001	[1.99, 7.42]	937	93.20	< 0.001
From special education, autism	0.17	0.04	< 0.001	[0.11, 0.26]	2.92	0.85	< 0.001	[1.65, 5.15]		137.99	< 0.001
Number of changes of school											
1–2	1.00	0.21	0.996	[0.66, 1.51]	0.55	0.12	0.006	[0.36, 0.84]	1186	13.16	< 0.001
≥ 3	0.56	0.13	0.015	[0.35, 0.89]	0.36	0.09	< 0.001	[0.22, 0.58]		4.67	0.031
Adequacy of support in school											
Never or rarely adequate	0.65	0.10	0.004	[0.48, 0.87]	0.64	0.10	0.006	[0.47, 0.88]	1247	0.00	0.978

Each analysis is adjusted for the age and sex of the individual with ASD. *RRR* relative risk ratio, *SE* standard error, *CI* confidence interval

Definition of variables and Reference Group (RG): parental highest education = primary/lower secondary school, upper secondary school, postsecondary/vocational education (RG); intellectual disability = present, not present (RG); part-time job = yes, no (RG); availability of current support = available, no but would like support, unnecessary (RG); population density = intermediate population density, thinly populated, densely populated (RG); compulsory schooling = special education, mainstream education (RG); schooling (hierarchical) = special (generic), special (autism), mainstream (RG); completion of lower secondary school = special education (generic), special education (autism), mainstream education (RG); number of changes of school = 1–2, 3– , 0 (RG); adequacy of support in school = never/rarely adequate, adequate/not necessary (RG)

Individuals with ID were significantly less likely to be in the group of normative daytime activity (RRR = 0.12) and significantly more likely to be in the group of customized daytime activity (RRR = 2.22) than in the group without regular daytime activity. Furthermore, individuals who had a part-time job at any point were 3.67 times more likely to be in the group in normative education/occupation than in the group without regular daytime activity. If support for the individual with ASD and/or the family was needed, individuals were significantly less likely to be in the group of normative education/occupation (support available RRR = 0.19; no support but would like support, RRR = 0.18) or in the group of customized daytime activity (support available RRR = 0.63; no support but would like support, RRR = 0.47) than in the group without regular daytime activity. However, the upper bound of the 95% confidence interval was very close to 1 for the group in customized education/occupation for “support available”, indicating a small possible effect.

With respect to the variables concerning type of schooling, it was found that individuals with ASD in normative education/occupation were significantly less likely to have attended school in special educational settings than were the individuals with ASD without regular daytime activity [compulsory schooling RRR = 0.38; schooling (hierarchical) RRR = 0.26 for generic special education and RRR = 0.25 for autism-specific special education; and school of completion RRR = 0.27 for generic special education and RRR = 0.17 for autism-specific special education]. Furthermore, the individuals with ASD in customized education/occupation were significantly more likely to have attended school in special educational settings than were the individuals with ASD without a regular daytime activity [compulsory schooling RRR = 1.89; schooling (hierarchical) RRR = 2.27 for generic special education and RRR = 2.44 for autism-specific special education; and school of completion RRR = 3.84 for generic special education and RRR = 2.92 for autism-specific special education]. Additionally, for the group in normative education/occupation versus the group in customized education/occupation, Wald tests showed significant differences between the RRRs for the variables of schooling. With respect to number of changes of school, the study found that when the number of changes of school was three or more compared with no changes, individuals with ASD were significantly less likely to be in the group of normative daytime activity (RRR = 0.56) or in the group of customized daytime activity (RRR = 0.36) than in the group without a regular daytime activity. Additionally, for adequacy of support in school, individuals with ASD whose support was rated as “never or rarely adequate” compared with “adequate or not necessary” were significantly less likely to be in the group of normative daytime activity (RRR = 0.65) or in the group of customized daytime activity (RRR = 0.64) than in the group without regular daytime activity.

Level of parental education (that is, highest educational level) was not significantly associated with group of daytime activity. With respect to population density, individuals with ASD in normative education/occupation were significantly less likely to be living in thinly populated areas (RRR = 0.58) than were the individuals with ASD without regular daytime activity. However, the remaining comparisons within population density were not significant.

## Discussion

This large, nationwide, Danish survey provides a description of the daytime activities performed by young adults diagnosed with ASD in childhood. Comparisons of behavioral parameters across groups based on current daytime activity were made, and associations between contextual factors primarily related to compulsory schooling and groups of daytime activity were investigated.

### Behavioral Characteristics and Comorbidity of the Groups of Daytime Activity

Differences in behavioral parameters were found when comparing the three groups of daytime activity in which the individuals with ASD were categorized. Adults in the group engaged in normative education/occupation were, on average, quite well functioning in different aspects, including having a near-normal score on the ABAS-II and low proportions of ID, current maladaptive behavior, and psychiatric comorbidity. Furthermore, this group had the lowest mean score on the RAADS-14 Screen of 19.8 versus the other groups studied, but note that 19.8 is above the cut-off score of 14 for screening for ASD suggested by Eriksson et al. (2013). However, the cut-off score should be interpreted with caution owing to a shift from self-report to parental report when using the RAADS-14 Screen in this study. Overall, the characteristics of adults in normative education/occupation, including higher functioning level, fewer ASD symptoms, and lower proportions of maladaptive behavior, are similar to what has been found in previous research (Roux et al. 2013; Shattuck et al. 2012; Taylor and Seltzer 2011; Taylor et al. 2015).

The proportion of lifetime maladaptive behavior did not differ between the group in normative education/occupation and the group in customized education/occupation. Hence, maladaptive behavior at any point in life in the population of individuals with ASD was frequent, regardless of current daytime activity. Concerning autism symptoms and adaptive behavior, similar levels of autism symptomatology and adaptive functioning were found for the group in customized education/occupation and the group without a regular daytime activity. However, a difference could have been expected for adaptive behavior owing to the difference across the groups in proportions of ID, which is known to affect adaptive behavior considerably (Kanne et al. 2011). The finding in our study that the presence of ID was not consistently a factor of importance in having a regular daytime activity or not is in line with the study by Taylor and Seltzer (2011). In the present study, the adults in customized education/occupation differed from the adults without a regular daytime activity in the proportions of current maladaptive behavior, anxiety, and depression, suggesting a higher well-being of the participants in the former group. This result was further supported by the quality of life (QoL) analyses performed by our research group on an almost identical study sample (Knüppel et al. 2018a). In this QoL study, being engaged in any type of occupation or being enrolled in any type of education was significantly associated with a higher level of QoL than being involved in no regular daytime activity. In contrast to the study by Taylor et al. (2014), the causal direction between the behavioral characteristics found in the group without a regular daytime activity and their daytime activity status could not be established. However, the overall results of the present study suggest that those in the group without a regular daytime activity had higher levels of behavioral difficulties and comorbidity than those in the groups in any education/occupation, indicating an urgent need for support of these young adults.

### Parental Evaluation of the Occupation of Their Adult Child

Importantly, in the present study, adults with ASD engaged in an occupation were, according to parental evaluation, generally satisfied with their occupation. There seemed to be a fit between the level of education of the adult with ASD and their occupation, which is in contrast to previous studies that found that individuals with ASD appeared to be overeducated for their jobs (Baldwin et al. 2014; Gotham et al. 2015). Furthermore, it was found that 9/10 of the adults engaged in vocational activities generally liked their occupation, and almost half were working full time or nearly full time (i.e., at least 30 h/week). However, it is important to note that this sample consisted of young adults, with the majority still enrolled in education with a pending future transition from education to occupation. This transition is known to be troublesome (Hendricks and Wehman 2009), which is acknowledged by individuals with ASD themselves (Van Hees et al. 2015). Hence, it is unknown whether, for example, underemployment according to educational level becomes a future problem when the young adults in this sample reach higher educational levels. Furthermore, even though adults with no regular daytime activity at the time of the study constituted approximately one-fifth of the sample, this might increase over time if a large group of young adults fails to move from education to employment.

### Individual and Contextual Factors Associated with Groups of Daytime Activity

Several factors were associated with the three groups of daytime activity. Young adults with ASD who had at any point in time had a part-time job were 3–4 times more likely to have a normative daytime activity than individuals without a regular daytime activity. This result is in line with previous research of young adults with disabilities (Carter et al. 2012; Test et al. 2009) and indicates, as also emphasized by Carter et al. (2012), that early work experiences are valuable. According to the present study, this also seems to be the case for individuals with ASD. However, this result was not found for individuals in customized education/occupation versus individuals without a regular daytime activity, suggesting that experiences from part-time work are not important for all types of education or occupation. Moreover, the present results suggest that adults without a regular daytime activity might not have access to the support/services they need, either currently or during compulsory school, and this might have had a negative impact on obtaining a daytime activity. Similarly, Ditchman, Miller, and Easton (2017) found that a higher number of services received by the individual with ASD was related to better employment outcome.

Concerning primary and lower secondary school, note that most of the present study population, regardless of current daytime activity, had at some point been schooled in special educational settings; however, tendencies in the data emerged. Adults with ASD were more likely to be in the group of normative education/occupation than in the group without a regular daytime activity when mainstream school had been the primary type of schooling, including type of school for completion. However, results favoring special educational settings were found for the group in customized education/occupation versus the group without regular daytime activity. Previously, studies have found varying results for the association between type of schooling and later daytime activity (Chan et al. 2017; Chiang et al. 2012; Foster and Pearson 2012), and the results from the present study indicate a complex relationship between type of schooling and later daytime activity for young adults diagnosed with ASD in childhood. Of note, the group of adults with ASD without a regular daytime activity had more often attended mainstream schooling than those in the group in customized education/occupation but less often than those in the group in normative education/occupation. Considering the higher number of school changes, individuals without a regular daytime activity might have been “stuck in the middle”, having difficulties too severe for mainstream education but possibly having been evaluated to have a functioning level too high for special educational settings. Currently, these young adults might have difficulties finding a relevant daytime activity.

To further study the association between daytime activity and type of schooling, it is also important to include factors such as the teacher’s knowledge of ASD and classroom intervention strategies for children with special needs in addition to measurements of the academic level, social inclusion, and well-being of the child with ASD. Such factors might clarify the overall effect of the type of schooling and provide knowledge about which children thrive and develop in various educational settings. As emphasized by Parsons (2015), appropriate education for some children and adolescents with ASD may be in special settings.

Contrary to existing research (Chan et al. 2017; Chiang et al. 2013), the parental level of education and the population density of the residence of the adults with ASD were not consistently associated with the three groups of daytime activity in this study. However, note that parental respondents to this survey were, to some extent, more highly educated than nonrespondents (Knüppel et al. 2018b), which might have affected the findings. However, in Denmark, for example, social and health services and education are free of charge, which might result in a high degree of social equality, hindering an association between parental level of education and group of daytime activity. Population density might affect daytime activity opportunities, as individuals with ASD living in more densely populated areas have a larger range of possible daytime activities available to choose from that match their needs. However, a possible association between population density and groups of daytime activity may not be identifiable for this study population due to the relatively uniform population density in a small country such as Denmark. Overall, this issue requires further investigation.

### Strengths and Limitations

The use of a large and nationwide sample is a major strength of this study. However, limitations do exist. First, it was not possible to verify the information provided by the parents, and recall bias may have arisen when parents were asked to provide historical information (e.g., schooling and behavioral difficulties). Second, the survey and register data on parental educational level and population density were not aligned in time, which may have affected the associations found. Third, the quality of ratings of the experience of being engaged in an occupation may have been improved if the adults with ASD answered those questions themselves. However, the number of questions directed to the individuals with ASD were kept at a minimum, as the level of functioning of the individuals with ASD was unknown before the survey launch. Finally, we had no information about whether distinctive characteristics, such as more severe intellectual and functional disability, were present in the children attending special education than in the children attending mainstream education. This may result in unequal benefits from education and affect the possibility of engaging in later daytime activity. Thus, this information could have clarified the results.

## Conclusions

This study investigated current daytime activity in a large, nationwide sample of young adults diagnosed with ASD in childhood. Approximately one-fifth of the sample did not have a regular daytime activity. Furthermore, this group differed from groups encompassing individuals in normative or customized education/occupation by having larger proportions of behavioral difficulties and psychiatric comorbidity. The adults with ASD in normative education/occupation were found to be well functioning in many aspects, with, for example, the highest level of adaptive behavior and the lowest proportion of overall psychiatric comorbidity. ID and part-time job were associated with group of daytime activity as well as with type of schooling during primary and lower secondary school, number of school changes, and type of schooling completed. However, different effects of these factors were found for different groups of daytime activity. Inadequacy or lack of support and services over time were associated with no engagement in regular daytime activities, indicating an unmet support need for a group of young adults with ASD.
